# Arthritis in the Canadian Aboriginal Population: North-South Differences in Prevalence and Correlates* *This article is part of a joint publication initiative between *Preventing Chronic Disease* and *Chronic Diseases in Canada*. *Preventing Chronic Disease* is the secondary publisher, while *Chronic Diseases in Canada* is the primary publisher.


**Published:** 2010-12-15

**Authors:** C. Ng, T. Kue Young, S. Chatwood

**Affiliations:** Institute of Medical Science, University of Toronto, Toronto, Ontario; Dalla Lana School of Public Health, University of Toronto, Toronto, Ontario; Institute for Circumpolar Health Research, Yellowknife, Northwest Territories. Dalla Lana School of Public Health, University of Toronto, Toronto, Ontario

## Abstract

**Background:**

Information on arthritis and other musculoskeletal disorders among Aboriginal people is sparse. Survey data show that arthritis and rheumatism are among the most commonly reported chronic conditions and their prevalence is higher than among non-Aboriginal people.

**Objective:**

To describe the burden of arthritis among Aboriginal people in northern Canada and demonstrate the public health significance and social impact of the disease.

**Methods:**

Using cross-sectional data from more than 29 000 Aboriginal people aged 15 years and over who participated in the Aboriginal Peoples Survey 2006, we assessed regional differences in the prevalence of arthritis and its association with other risk factors, co-morbidity and health care use.

**Results:**

The prevalence of arthritis in the three northern territories ("North") is 12.7% compared to 20.1% in the provinces ("South") and is higher among females than males in both the North and South. The prevalence among Inuit is lower than among other Aboriginal groups. Individuals with arthritis are more likely to smoke, be obese, have concurrent chronic diseases, and are less likely to be employed. Aboriginal people with arthritis utilized the health care system more often than those without the disease.

**Conclusion:**

Aboriginal-specific findings on arthritis and other chronic diseases as well as recognition of regional differences between North and South will enhance program planning and help identify new priorities in health promotion.

**Keywords:**

arthritis, Aboriginal people, Northern Canada, Inuit, First Nations, Métis, North American Indians, Aboriginal Peoples Survey

## Introduction

Information on arthritis and other musculoskeletal disorders among Aboriginal people is sparse and geographically limited—mainly to Alaska, British Columbia and Manitoba.^1-3^ Several national surveys—the First Nations Regional Longitudinal Health Survey (RHS),^4,5^ the Canadian Community Health Survey (CCHS)^6-7^ and the Aboriginal Peoples Survey (APS)^8-9^—have provided some data on the prevalence of arthritis, rheumatism and other musculoskeletal conditions, such as back pain, among adults in the Aboriginal population. These surveys generally show that arthritis and rheumatism are among the most commonly reported chronic conditions, that prevalence is higher than among non-Aboriginal people in Canada, and that prevalence is increasing; for example, the crude prevalence was 15% in 1991 and 19% in 2001 according to the APS,^8,9^ while the age-adjusted prevalence was 22% in 1997 and 25% in 2002/03 according to the RHS.^4,5^ (Note that these surveys are not directly comparable with one another due to the inclusion of different Aboriginal groups and the use of different standard populations in age-adjustment of rates.) Arthritis also contributes to more than half of the self-reported disability among First Nations people in Canada.^5^

Disability resulting from arthritis can be exacerbated in the north of Canada by severe weather, inadequate infrastructure and unreliable transportation. Arthritis compromises the ability of Aboriginal people to pursue traditional activities, such as harvesting country foods, and traditional crafts. The geographical isolation of many communities reduces access to specialist services. Cultural context is an additional dimension and requires region-specific directions and broad partnerships to plan and implement culturally appropriate health services and support systems; specific considerations include, but are not limited to, access to traditional healers and medicines, languages spoken, and the design of support services in communities.

This paper describes the burden of arthritis among Aboriginal people in Yukon, Northwest Territories and Nunavut—the three northern territories of Canada. We assess regional differences in the prevalence of arthritis and its association with other risk factors, co-morbidity and health care use between these three northern territories (the "North") and the ten provinces of southern Canada (the "South") using data from the recently released APS 2006.^10^

## Methods

We used cross-sectional data from more than 29 000 Aboriginal respondents (off-reserve First Nations, Métis, Inuit) aged 15 years and over who participated in the APS 2006 (Table 1). Statistics Canada conducted the APS as a post-censal survey to collect information on the social and economic conditions of Aboriginal people living in Canada.

The APS 2006 asked respondents whether a doctor, nurse or other health professional had ever told them that they have arthritis or rheumatism. We separately examined associations between arthritis and various demographic, socioeconomic, behavioural and health care correlates for the three territories and the 10 provinces, not to test specific etiological hypotheses but to demonstrate the public health significance and social impact of the burden of arthritis. Obesity, defined as body mass index (BMI) of 30 kg/m^2^ or higher, and smoking are well-established risk factors for arthritis,^11,12^ and the APS 2006 asked respondents about their height, weight and smoking experience and habits. Arthritis is also associated with reduced employment and work limitations among adults;^13^ the APS asked respondents, "Last week, did you work for pay or in self-employment?"

We determined prevalence of arthritis for three separate groups based on the question "Do any of your ancestors belong to the following Aboriginal groups? (Can check more than one): North American Indian, Métis or Inuit." Individuals who checked only "North American Indian" constitute the "First Nations" group, individuals who checked only "Inuit" constitute the "Inuit" group, and all others including Métis and those who checked multiple Aboriginal groups were combined into an "Other" cate­gory as each of these groups have small sample sizes in the North. We report only crude prevalence proportions; we did not compute age-adjusted prevalence as the dataset did not include non-Aboriginal people for comparison, and comparing this study with published age-adjusted rates is difficult due to the different standard popu­lations that have been used; further, the crude prevalence more accurately reflects the burden of disease needed to plan public health programs.

We performed all analyses using SAS version 9.2 (SAS Institute Inc, Cary, North Carolina). Since the APS 2006 was based on a complex survey design, we used survey weights in all analyses and calculated variance estimates using the bootstrap technique with the 1000 bootstrap weights provided by Statistics Canada. We determined all proportions in accordance with rounding guidelines suggested by Statistics Canada and calculated confidence intervals (CIs) from unrounded components. Detailed survey methodology is available from Statistics Canada.^10^ We used age- and sex-adjusted logistic regression analyses to assess associations between arthritis and various correlates.

## Results

### Prevalence

The crude prevalence of arthritis or rheumatism for the three combined Aboriginal groups in the territories is 12.7% (95% CI: 12.5-13.0) compared to 20.1% in the provinces (95% CI: 19.9-20.3). Arthritis is more prevalent among females than males in both the North and South. The prevalence among Inuit is lower than among First Nations and other Aboriginal groups. As expected, prevalence increases with age (Table 2).

### Health risks

For a comparison of the proportion of respondents with and without arthritis who are daily smokers, are obese or have co-morbid conditions, see Table 3.

Smoking is more prevalent among Aboriginal people in the North than in the South. In the South, there is an association between daily smoking and arthritis (age-sex-adjusted odds ratio [OR] = 1.58, 95% CI: 1.53-1.62), but daily smoking is not a significant factor in the North (OR = 1.05, 95% CI: 0.98-1.12). Obesity is more prevalent among individuals with arthritis, and the association between obesity and arthritis is stronger among Aboriginal people in the South (OR = 1.59, 95% CI: 1.54-1.64) than in the North (OR = 1.36, 95% CI: 1.26-1.47).

In both the South and the North, a higher proportion of individuals with arthritis than those without report having at least one other chronic condition such as diabetes, heart disease, hypertension, stroke, asthma, chronic bronchitis, emphysema or cancer (for the North, OR = 1.88, 95% CI: 1.77-2.00; for the South, OR = 2.55, 95% CI: 2.48-2.61).

### Health care use

**Figure 1:**
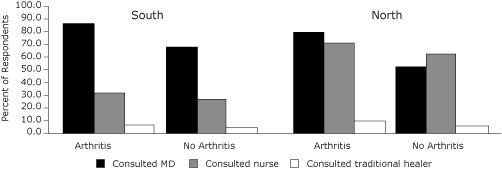
Utilization of health services by Aboriginal people aged 15 years and over in the North a and South^b^ of Canada by type of provider and by arthritis status.

The proportion of individuals who report consulting a health professional (primary care physician or nurse) or traditional healer† anytime in the 12 months preceding the survey was higher among individuals with arthritis than those without the condition. (In the North, OR = 2.32, 95% CI: 2.10-2.56; in the South, OR = 2.25, 95% CI: 2.17-2.33.) In the North, arthritis patients consulted nurses and traditional healers more and physicians less frequently than those in the South. (See Figure 1)

**Table:** 

	**South**				**North**			

	**Arthritis**		**No Arthritis**		**Arthritis**		**No Arthritis**	

	**%**	**95% CI**	**%**	**95% CI**	**%**	**95% CI**	**%**	**95% CI**
Consulted MD	86.0	85.7-86.4	67.6	67.4-67.8	79.0	78.0-79.9	52.3	51.8-52.7
Consulted nurse	31.4	30.9-31.9	26.9	26.6-27.1	71.0	69.9-72.2	62.3	61.9-62.8
Traditional healer	6.7	6.4-6.9	4.4	4.3-4.5	9.6	8.7-10.4	5.8	5.5-6.1

a The three Canadian northern territories: Yukon, Northwest Territories, Nunavut.

b The 10 Canadian provinces: British Columbia, Alberta, Saskatchewan, Manitoba, Ontario, Quebec, New Brunswick, Prince Edward Island, Nova Scotia, Newfoundland and Labrador.

† In the APS 2006, a traditional healer refers to someone who is recognized by the community as a traditional counsellor, or someone who provides traditional medicines such as herbs, or is a traditional or spiritual leader.

### Social conditions

A lower proportion of individuals with arthritis report being employed in the week before the survey (either self-employed or otherwise working for pay) compared to those without arthritis. The association was stronger in the South (OR = 0.58, 95% CI: 0.57-0.59) than in the North (OR = 0.75, 95% CI: 0.70-0.79).

## Discussion

The lower prevalence estimates among Aboriginal people in the North compared to those in the South obtained from the APS 2006 are comparable to those from other surveys.^6,7^ In Tjepkema's analyses of CCHS 2000/01,^6^ the prevalence for Aboriginal people in the North is 10% while that in the South is 19% for rural residents and 20% for urban residents; Lix et al. obtain a prevalence of 12% in the North and 20% in the South from the CCHS 2005/06.^7^ Both these studies also show that the prevalence among Aboriginal people is higher than non-Aboriginal people in the South but not in the North. Note that both the CCHS and APS cover the same Aboriginal groups—off-reserve First Nations, Inuit and Métis. Although less access to specialist care may be responsible for the lower detection rate of arthritis in the North, the prevalence of arthritis is based on self-report and not on clinically verified diagnoses by rheumatologists; further, as a chronic disease arthritis is likely to have been diagnosed sometime in the past over the long term even with limited specialist health care.

In surveys such as the APS, CCHS and RHS, self-reports under the rubric "arthritis and rheumatism" lack clinical accuracy. These self-reports are also limited by the inability to differentiate between different types of arthritides—rheumatoid arthritis, osteoarthritis, etc. However, as a tool for assessing population health and the need for health care, such crude measures are nevertheless useful, particularly to describe the patterns in different population subgroups.

The lower prevalence of arthritis among Aboriginal people in the North can also be attributed to the high proportion of Inuit in the population. (According to the 2006 Census, approximately 54% of Aboriginal residents of the northern territories report some Inuit ancestry, compared to 4% of Aboriginal people in Canada as a whole.^14^) A lower prevalence of arthritis among Inuit relative to other Aboriginal people has been shown nationally in APS 2001^9^ and CCHS 2000/01.^6^ In this study we demonstrate that, within the North, the prevalence of arthritis among all Aboriginal groups—Inuit, First Nations, and Other—is also lower than the corresponding group in the South (Table 2).

It is unclear as to why Canadian Inuit have lower prevalence of arthritis than First Nations people. The self-reported arthritis rubric is a mixed bag of clinical entities with different etiologies. A review of North American indigenous populations found that Inuit tend to have high rates of spondyloarthropathies whereas Native Americans have high rates of rheumatoid arthritis.^1^ A study based on clinical records indicates that the Inupiat in the Alaska North Slope region (who are culturally and linguistically related to the Inuvialuit in the Northwest Territories) have high rates of rheumatoid arthritis compared to some Native American tribes, and much higher than the Yupik in western Alaska.^15^ A recent study from Alaska that estimated the prevalence of self-reported and clinically undifferentiated arthritis showed that it is higher among Alaska Natives than the general U.S. population, but the Alaskan sample is a mix of Yupik and Native American tribes in the southeastern part of the state.^16^

Aboriginal people suffering from arthritis have unfavourable health profiles; they are more likely to be daily smokers, be obese and have concurrent chronic diseases, although the magnitude differs between the North and South, reflecting the background prevalence of these associated traits and conditions. Arthritis can limit the opportunity for employment, although this survey does not provide evidence that the lower employment rate is the direct result of the disease.

As expected, Aboriginal people with arthritis are more likely to utilize the health care system, with higher proportions reporting visits to physicians, nurses and traditional healers. The pattern of use reflects the different systems in place in the North and South. We cannot, however, determine if the higher health service use is the direct result of arthritis, but it is a plausible explanation given the nature of the disease, the presence of other risk factors such as smoking and obesity, and co-morbidities. In the North, primary care is predominantly delivered by nurses in health centres in the communities, and individuals have only periodic contact with visiting physicians. For many, visits to specialists such as rheumatologists requires air travel away from home.

Further research is required to explore North-South disparities in the burden of arthritis in Aboriginal populations. Also needed are more refined diagnoses, including rheumatoid arthritis, osteoarthritis and other musculoskeletal disorders, as well as separate analyses of Inuit and First Nations samples, which are sufficiently large within the North. Aboriginal-specific findings on arthritis and other chronic diseases, as well as recognition of regional differences between North and South, will enhance program planning and help identify new priorities in health promotion. The creation and transmission of quality evidence to appropriate stakeholders to ensure uptake and application of study findings will help reduce health disparities.

## Figures and Tables

**Table 1:** Number of respondents aged 15 years and over by geographic region and Aboriginal group in the North^a^ and South^b^ of Canada

	North^a^		South^b^	

	Male	Female	Male	Female
First Nations	380	420	5260	6680
Inuit	1630	1650	830	880
Other^c^	270	260	4990	5870

Note: numbers are unweighted and have been rounded to the nearest ten.

a The three Canadian northern territories: Yukon, Northwest Territories, Nunavut.

b The 10 Canadian provinces: British Columbia, Alberta, Saskatchewan, Manitoba, Ontario, Quebec, New Brunswick, Prince Edward Island, Nova Scotia, Newfoundland and Labrador.

c Respondents of Métis or multiple Aboriginal ancestry.

**Table 2:** Crude prevalence (%) of arthritis and rheumatism among Aboriginal people aged 15 years and over in the North^a^ and South^b^ of Canada

	North^a^			South^b^		

	Male	Female	Both	Male	Female	Both

	Prevalence (%)(95% CI)	Prevalence (%)(95% CI)	Prevalence (%)(95% CI)	Prevalence (%)(95% CI)	Prevalence (%)(95% CI)	Prevalence (%)(95% CI)
All Aboriginal groups	10.2 (9.8-10.6)	15.2 (14.7-15.6)	12.7 (12.5-13.0)	16.2 (16.0-16.5)	23.3 (23.0-23.5)	20.1 (19.9-20.3)
First Nations	12.0 (11.1-12.8)	16.0 (15.1-16.9)	14.2 (13.5-14.8)	14.9 (14.6-15.3)	23.2 (22.9-23.6)	19.6 (19.3-19.8)
Inuit	8.8 (8.4-9.2)	14.5 (14.0-15.0)	11.7 (11.4-12.0)	9.7 (8.7-10.8)	18.3 (16.9-19.7)	14.0 (13.1-14.9)
Other	10.7 (9.6-11.8)	15.4 (14.2-16.6)	12.9 (12.1-13.8)	18.0 (17.7-18.4)	23.5 (23.2-23.9)	21.0 (20.7-21.2)
15-24 years	1.8 (1.5-2.1)	2.6 (2.3-3.0)	2.2 (2.0-2.4)	3.0 (2.7-3.2)	4.5 (4.2-4.8)	3.8 (3.5-4.0)
25-44 years	6.7 (6.3-7.2)	10.6 (9.9-11.2)	8.7 (8.3-9.1)	10.5 (10.2-10.8)	15.0 (14.7-15.4)	13.1(12.9-13.3)
45-64 years	20.9(19.7-22.0)	29.5 (28.3-30.6)	25.3 (24.5-26.1)	29.0 (28.4-29.6)	40.0(39.5-40.6)	35.0 (34.6-35.4)
65+ years	34.4 (32.0-36.7)	51.8 (49.3-54.3)	43.5 (41.8-45.2)	40.1 (39.0-41.3)	60.8 (59.7-61.9)	51.8 (50.9-52.6)

Abbreviations: CI, confidence interval.

a The three Canadian northern territories: Yukon, Northwest Territories, Nunavut.

b The 10 Canadian provinces: British Columbia, Alberta, Saskatchewan, Manitoba, Ontario, Quebec, New Brunswick, Prince Edward Island, Nova Scotia, Newfoundland and Labrador.

**Table 3:** Crude prevalence (%) of health risks associated with arthritis among Aboriginal people aged 15 years and over in the North^a^ and South^b^ of Canada

	North^a^		South^b^	

	Arthritis	No Arthritis	Arthritis	No Arthritis

	Prevalence (%)(95% CI)	Prevalence (%)(95% CI)	Prevalence (%)(95% CI)	Prevalence (%)(95% CI)
Daily smoking	44.8 (43.6-46.1)	51.6 (51.1-52.1)	36.2 (35.8-36.7)	29.6 (29.4-29.8)
Obese (BMI = 30)	36.7 (35.4-38.0)	23.7 (23.2-24.2)	33.4 (32.9-33.8)	22.6 (22.4-22.9)
Co-morbid conditions^c^	47.0 (45.8-48.2)	20.4 (20.0-20.8)	61.6 (61.1-62.1)	29.1 (28.9-29.3)

Abbreviations: BMI, body mass index; CI, confidence interval.

a The three Canadian northern territories: Yukon, Northwest Territories, Nunavut.

b The 10 Canadian provinces: British Columbia, Alberta, Saskatchewan, Manitoba, Ontario, Quebec, New Brunswick, Prince Edward Island, Nova Scotia, Newfoundland and Labrador.

c Co-morbid conditions include diabetes, hypertension, heart disease, asthma, chronic bronchitis, emphysema, cancer and stroke.

## References

[1] Peschken CA, Esdaile JM (1999). Rheumatic diseases in North America's indigenous peoples. Semin Arthritis Rheum.

[2] Barnabe C, Elias B, Bartlett J, Roos L, Peschken C (2008). Arthritis in Aboriginal Manitobans: evidence for a high burden of disease. J Rheumatol.

[3] Ferucci ED, Templin DW, Lanier AP (2005). Rheumatoid arthritis in American Indians and Alaska Natives: a review of the literature. Semin Arthritis Rheum.

[4] Young TK, O'Neil JD, Elias B, Leader A, Reading J, McDonald G, Walsh C, O'Neil JD, Jamieson E, Crawford A, Boyle M Chapter 3: Chronic diseases. First Nations and Inuit Regional Health Survey Final Report [Internet].

[5] Assembly of First Nations/First Nations Information Governance Committee (2007). First Nations Regional Longitudinal Health Survey (RHS) 2002/2003: results for adult, youth and children living in First Nations communities. Rev. 2nd ed..

[6] Tjepkema M (2002). The health of the off-reserve Aboriginal population. Health Rep.

[7] Lix LM, Bruce S, Sarkar J, Young TK (2009). Risk factors and chronic conditions among Aboriginal and non-aboriginal populations. Health Rep.

[8] Statistics Canada (1993). Language, tradition, health, lifestyle and social issues: 1991 Aboriginal Peoples Survey.

[9] O'Donnell V, Tait H (2003). Aboriginal Peoples Survey 2001 - initial findings: well-being of the non-reserve Aboriginal Population.

[10] Statistics Canada (2009). Aboriginal Peoples Survey, 2006: concepts and methods guide.

[11] Oliveria SA, Felson DT, Cirillo PA, Reed JI, Walker AM (1999). Body weight, body mass index, and incident symptomatic osteoarthritis of the hand, hip, and knee. Epidemiology.

[12] Heliovaara M, Aho K, Aromaa A, Knekt P, Reunanen A (1993). Smoking and risk of rheumatoid arthritis. J Rheumatol.

[13] Lacaille D, Hogg RS (2001). The effect of arthritis on working life expectancy. J Rheumatol.

[14] Statistics Canada (2009). Aboriginal ancestry (10), area of residence (6), age groups (12) and sex (3) for the population of Canada, provinces and territories, 2006 Census – 20% Sample.

[15] Boyer GS, Benevolenskaya LI, Templin DW, Erdesz S, Bowler A, Alexeeva LI, Goring WP, Krylor MY, Mylov NM (1998). Prevalence of rheumatoid arthritis in circumpolar native populations. J Rheumatol.

[16] Ferucci ED, Schumacher MC, Lanier AP, Murtagh MA, Edwards S, Helzer LJ, Tom-Orme L, Slattery ML (2008). Arthritis prevalence and associations in American Indian and Alaska Native people. Arthritis Rheum.

